# p27^Kip1^ regulates alpha-synuclein expression

**DOI:** 10.18632/oncotarget.24687

**Published:** 2018-03-27

**Authors:** Edurne Gallastegui, Carla Domuro, Joan Serratosa, Alejandra Larrieux, Laura Sin, Jonatan Martinez, Arnaud Besson, José Manuel Morante-Redolat, Serena Orlando, Rosa Aligue, Isabel Fariñas, María Jesús Pujol, Oriol Bachs

**Affiliations:** ^1^ Department of Biomedical Sciences, (CIBERONC), University of Barcelona, Barcelona, Spain; ^2^ Department of Cerebral Ischemia and Neurodegeneration, Institut d’Investigacions Biomèdiques de Barcelona-Consejo Superior de Investigaciones Científicas, Barcelona, Spain; ^3^ Cancer Research Center of Toulouse, Université Toulouse III Paul Sabatier, Toulouse, France; ^4^ Departamento de Biología Celular, Biología Funcional y Antropología Física, ERI de Biotecnología y Biomedicina, (CIBERNED), Universidad de Valencia, Valencia, Spain

**Keywords:** p27Kip1, p21Cip1, E2F4, alpha synuclein, transcription

## Abstract

Alpha-synuclein (α-SYN) is the main component of anomalous protein aggregates (Lewy bodies) that play a crucial role in several neurodegenerative diseases (synucleinopathies) like Parkinson’s disease and multiple system atrophy. However, the mechanisms involved in its transcriptional regulation are poorly understood. We investigated here the role of the cyclin-dependent kinase (Cdk) inhibitor and transcriptional regulator p27^Kip1^ (p27) in the regulation of α-SYN expression. We observed that selective deletion of p27 by CRISPR/Cas9 technology in neural cells resulted in increased levels of α-SYN. Knock-down of the member of the same family p21^Cip1^ (p21) also led to increased α-SYN levels, indicating that p27 and p21 collaborate in the repression of α-SYN transcription. We demonstrated that this repression is mediated by the transcription factor E2F4 and the member of the retinoblastoma protein family p130 and that it is dependent of Cdk activity. Chromatin immunoprecipitation analysis revealed specific binding sites for p27, p21 and E2F4 in the proximal α-SYN gene promoter. Finally, luciferase assays revealed a direct action of p27, p21 and E2F4 in α-SYN gene expression. Our findings reveal for the first time a negative regulatory mechanism of α-SYN expression, suggesting a putative role for cell cycle regulators in the etiology of synucleinopathies.

## INTRODUCTION

p27Kip1 (p27) encoded by the CDKN1B gene belongs to the Cip/Kip family of inhibitors of cyclin dependent kinases (Cdks) [1]. Cdks are a family of kinases involved in the regulation of the cell division cycle whose activity is regulated by a number of mechanisms that include cyclin association, phosphorylation, acetylation and binding to inhibitory proteins [2–5]. In addition to p27, the Cip/Kip family of Cdk inhibitors includes p21(Cip1) and p57(Kip2). Classically, the role of p27 was to inhibit the activity of cyclin- Cdk complexes by associating with both the cyclin and the Cdk. This association is performed by specific regions located at the NH2 terminus of the protein. The inhibitory effect is caused by the introduction of a specific small region of p27 inside of the catalytic cleave of the Cdk [2]. However, recent work has described that specific phosphorylations in tyrosine residues of p27 located in a domain, close to that interacting with the catalytic center of the Cdk, induce conformational changes in p27 leading to a decrease in its Cdk inhibitory capacity. Specifically, phosphorylations in Y74, Y88 and Y89 of p27 by different tyrosine kinases, mostly of the Src family, reduce the inhibition of Cdk activity by p27, despite p27 remains associated with the complex [6–8]. Thus, now, the concept is that p27 is not strictly a Cdk inhibitor but it is a Cdk regulator that when associated with cyclin-Cdk complexes might or not inhibit Cdk activity on depending on its phosphorylation status. This is compatible with previous reports showing Cdk4 activity in ternary p27-cyclin D3-Cdk4 complexes [9]. p27 has a relatively long carboxyl-moiety that has been identified as an intrinsically disordered region [10]. It means that it can adopt multiple conformations that confer to this region the capacity to interact with a significant number of different protein partners.

In addition to its role as a regulator of cyclin-Cdk activity, recent works have identified a new function of p27 as a transcriptional regulator [11]. Since it is not able to directly interact with DNA, its capability to behave as a transcriptional regulator is mediated by the association with a number of regulatory proteins as transcription factors (TFs), co-activators or co-repressors. Several reports by our group have revealed that p27 mostly interacts with distant intergenic and intronic regions of the chromatin although it can also associate with specific gene promoters [11–13]. p27 has been shown to interact with TFs as E2F4, Ets-1 and PAX5 and with the transcriptional co-activator (with acetyltransferase activity) PCAF [11, 13, 14]. Our group has been intensively working in the mechanisms of how p27 regulates the expression of E2F4 repressed genes. E2F4 participates in transcriptional repressor complexes that include p130 (a member of the retinoblastoma family) and other transcriptional co-repressors as several HDAC (histone deacetylases) and mSIN3A [11]. These complexes are specifically relevant because they associate to promoters of genes needed for DNA replication during the G1 phase of the cell cycle and repress their expression. p27 is able to directly associate with E2F4 and p130 by its carboxyl-terminal domain and in such a way it participates in the repressor function of these complexes [11]. Interestingly, during early and mid G1 phase of the cell cycle p27, associated with p130/E2F4 on the promoters, recruits cyclin D2/D3-Cdk4 complexes by its NH2 domain. Since p130 is a substrate of Cdk4, the role of p27 is to bring near p130 the kinase Cdk4 that after its subsequent activation will phosphorylate p130. At late G1, p27-cyclin D2/3 and Cdk4 are released from the p130/E2F4 complexes and are substituted by p21-cyclin D1-Cdk2 that by phosphorylating p130 remove these repressor complexes from the gene promoters allowing transcription of these genes [15]. Thus, in such a way, p27 and p21 collaborate in the transcriptional regulation of genes repressed by p130/E2F4 complexes. Expression of important genes, as for instance SOX2 involved in pluripotency, are also regulated by this mechanism [16, 17]. Recent reports also revealed that p27 collaborates with PAX5 and the transcriptional co-activator PCAF in the regulation of a number of genes involved in different cellular functions [14].

Expression microarray analysis performed in different cellular types have allowed the identification of the transcriptional programs regulated by p27 [11, 13, 18]. Interestingly, in addition to the expected involvement of p27 in the regulation of cell cycle, it also participates in the regulation of the expression of genes involved in transcription, splicing and respiration, among others [11]. Since low levels of p27 in tumors have been associated with a worse outcome of patients [19–21], it has been postulated that deregulation of these transcriptional programs in low-p27 tumors play an important role in their progression to malignancy [11].

Surprisingly, expression microarray analysis performed in mice embryonic fibroblasts (MEFs) from p27 knock out (p27KO) animals versus control revealed that a significant number of genes involved in Parkinson’s disease (PD) were significantly deregulated [18]. These genes are involved in the induction of the three main Hallmarks of PD: Lewy pathology (accumulation and α-synuclein (α-SYN) aggregates), mitochondrial dysfunction and neuro-inflammation. It merits to emphasize that in p27KO-MEFs α-SYN mRNA is increased more than 8 fold. Since a neuropathological hallmark of PD and other α-synucleinopathies is the intracellular accumulation of misfolded α-SYN we aimed to analyze the putative role of p27 in the regulation of α-SYN expression. Despite the intense debate of how α-SYN pathology initiates and spreads in the nervous system, the mechanisms involved in the regulation of α-SYN expression still remain largely unknown. We report here that α-SYN transcription is regulated by p27 in collaboration with p21, mediated by p130/E2F4 complexes and depending of Cdk activity. These results suggest a putative involvement of p27 in the etiology of PD and perhaps other α-synucleinopathies.

## RESULTS

### p27 and p21 regulate the expression of α-SYN in c17.2 neural stem cells

**Figure 1:**
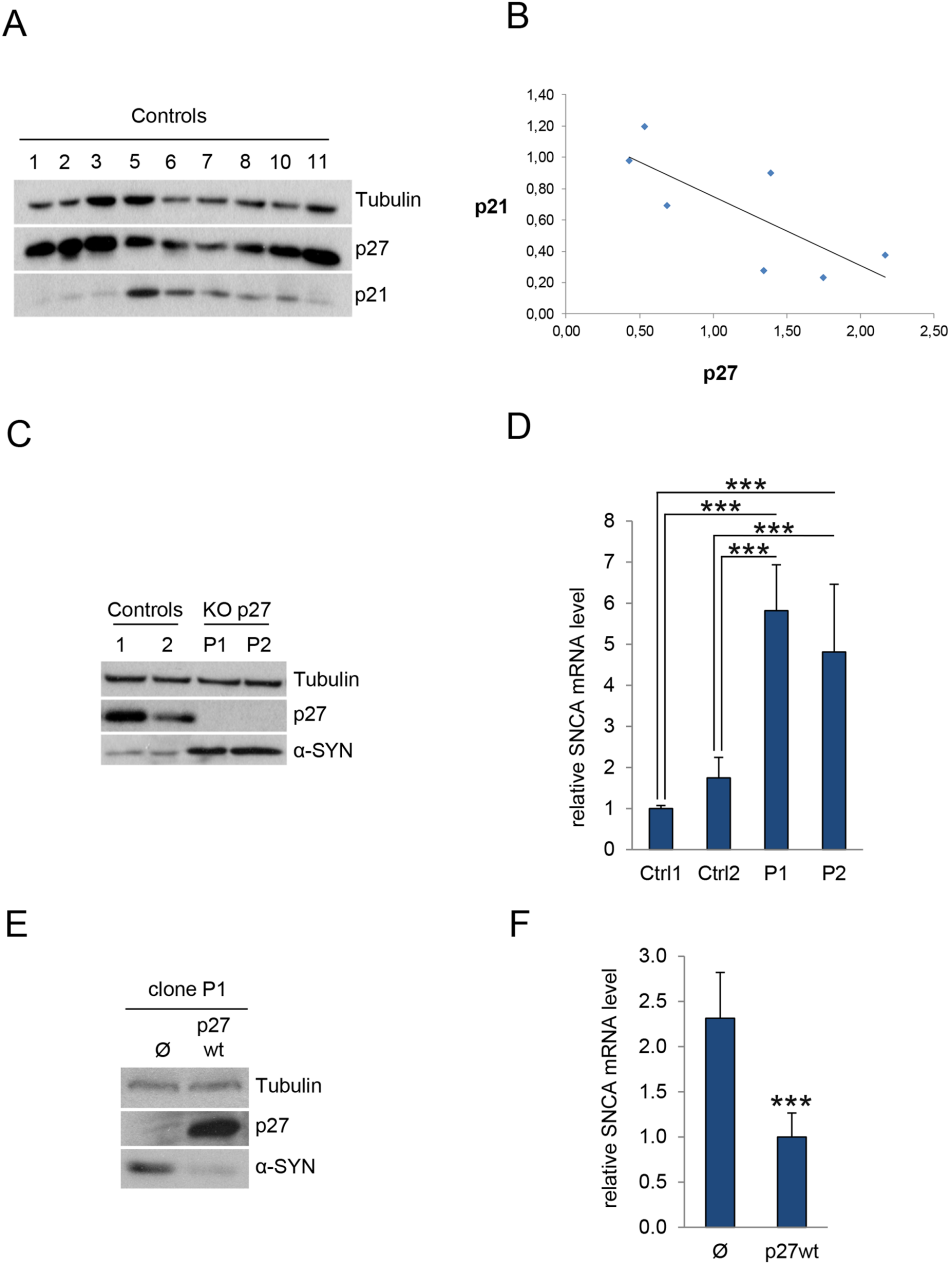
p27 regulates α-SYN expression **(A)** c17.2 cells were knocked out for p27 by using the CRISPR/Cas9 methodology. Then, severalcontrol c17.2 clones were assessed for p27 and p21 by WB. Tubulin was used as a loading control. **(B)** The bands from Figure 1A were quantified by using the image J program. After normalizing data respect tubulin, a correlation between p27 and p21 levels was analyzed using the regression lineal program SPSS. The significance was P<0.05. The value of the Pearson correlation was -0,762. **(C)** The levels of p27 and α-SYN were determined by WB in two control clones (1 and 2) and two p27KO clones (P1 and P2). Tubulin was used as a loading control. **(D)** Levels of α-SYN mRNA were determined in the same samples by qPCR. Results are expressed relative to Control 1 (Ctrl1). **(E)** Cells from the p27KO clone P1 were infected with lentiviruses with an empty vector (Ф) or with wild-type p27 (p27wt). Then, protein levels of p27 and α-SYN protein were determined by WB. Tubulin was used as a loading control. **(F)** α-SYN mRNA levels were determined by qPCR in the same cells as in (E). In all experiments results are the mean value ± SD of at least three independent experiments. Statistical analyses were performed using the Student’s t-test. ^***^P<0.001.

Previous expression microarray analyses revealed an 8-fold increase of α-SYN mRNA in p27KO MEFs, suggesting that p27 might be involved in the transcriptional regulation of this gene [18]. To study this possibility, we generated a p27 deletion in c17.2 neural stem cells [22] using the CRISPR/Cas9 methodology. Next we selected different clones in which p27 was effectively deleted. Because we had reported that in MEFs, p27 regulates the expression of p21 [18] and, as aforementioned, p21 can collaborate with p27 in the regulation of different p130/E2F4 repressed genes [15], we first checked whether in c17.2 wt clones there was a correlation between p27 and p21 protein levels. As expected, we observed an inverse correlation of p27 and p21 in these clones. It can be seen that the clones with higher levels of p27 display lower amounts of p21 and vice versa. (Figure 1A and 1B).

**Figure 2:**
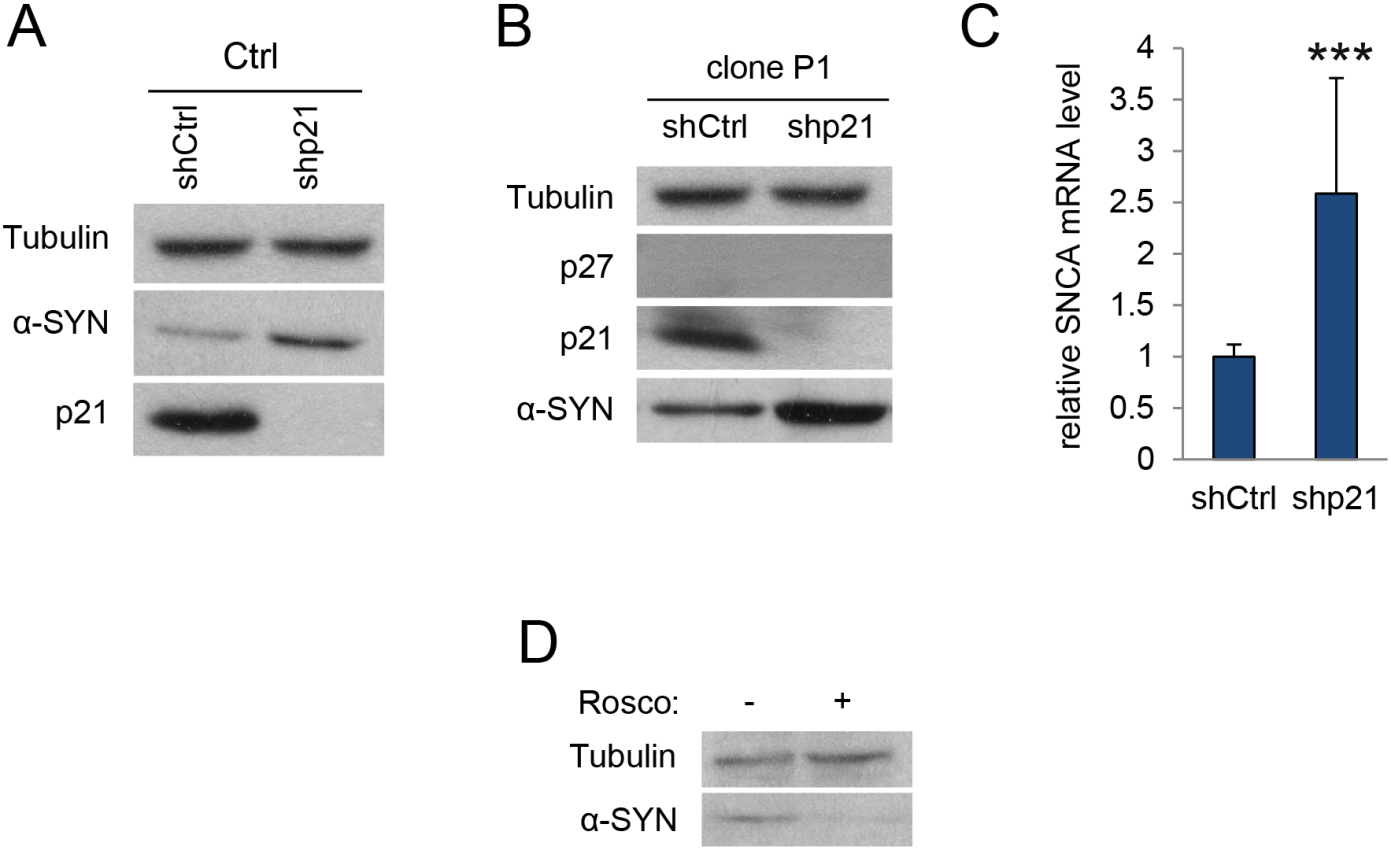
p21 regulates α-SYN expression **(A)** Levels of α-SYN and p21 were determined in c17.2 control cells infected with ShRNA control or ShRNA for p21. Tubulin was used as a loading control. **(B)** Cells from the p27KO clone P1 were infected with ShRNA control or ShRNA for p21 and subsequently assessed for p27, p21 and α-SYN protein by WB. Tubulin was used as a loading control. **(C)** α-SYN mRNA levels were determined by qPCR in the same cells as in (B). **(D)** α-SYN protein levels were determined in c17.2 cells treated or not with the Cdk inhibitor Roscovitine (20 μM). In all experiments results are the mean value ± SD of at least three independent experiments. Statistical analyses were performed using the Student’s t-test. ^***^P<0.001.

We then quantified the levels of α-SYN in the p27-null cells. WB analysis revealed that elimination of p27 resulted in increased amount of α-SYN (Figure 1C). Accordingly, we could also detect elevated levels of α-SYN mRNA in these cells (Figure 1D). To evaluate whether this was specifically mediated by p27, we re-introduced a wt version of p27 in p27-CRISPered cells. Overexpression of p27 in these cells rescued the phenotype and decreased both the α-SYN protein (Figure 1E) and mRNA levels (Figure 1F). On the other hand, to examine the possibility of an additional role of p21 in the regulation of α-SYN expression we knocked down (KD) p21 in a control clone with specific shRNAs. As observed in Figure 2A, decreasing p21 induces the elevation of α-SYN. Finally, we introduced the p21 shRNA into a p27KO clone and evaluated the effect on α-SYN expression finding that both α-SYN protein (Figure 2B) and mRNA (Figure 2C) were further increased. Together, all these results suggest that p27, in collaboration with p21, regulates α-SYN expression.

As p27 and p21 are classical inhibitors of different cyclin-Cdk complexes, the absence of these proteins induces the increase of Cdk activity that, according to our model of transcriptional regulation, will induce the expression of target genes by phosphorylating specific transcriptional regulators, as for instance p130. Thus, we aimed to analyze here the effect of the Cdk inhibitor Roscovitine on α-SYN expression in c17.2 cells. As it can be seen in Figure 2D, Roscovitine reduces the α-SYN levels in the cells, indicating that cyclin-Cdk complexes participate in the regulation of α-SYN expression.

### p27 regulation of α-SYN expression is mediated by p130/E2F4 complexes

**Figure 3:**
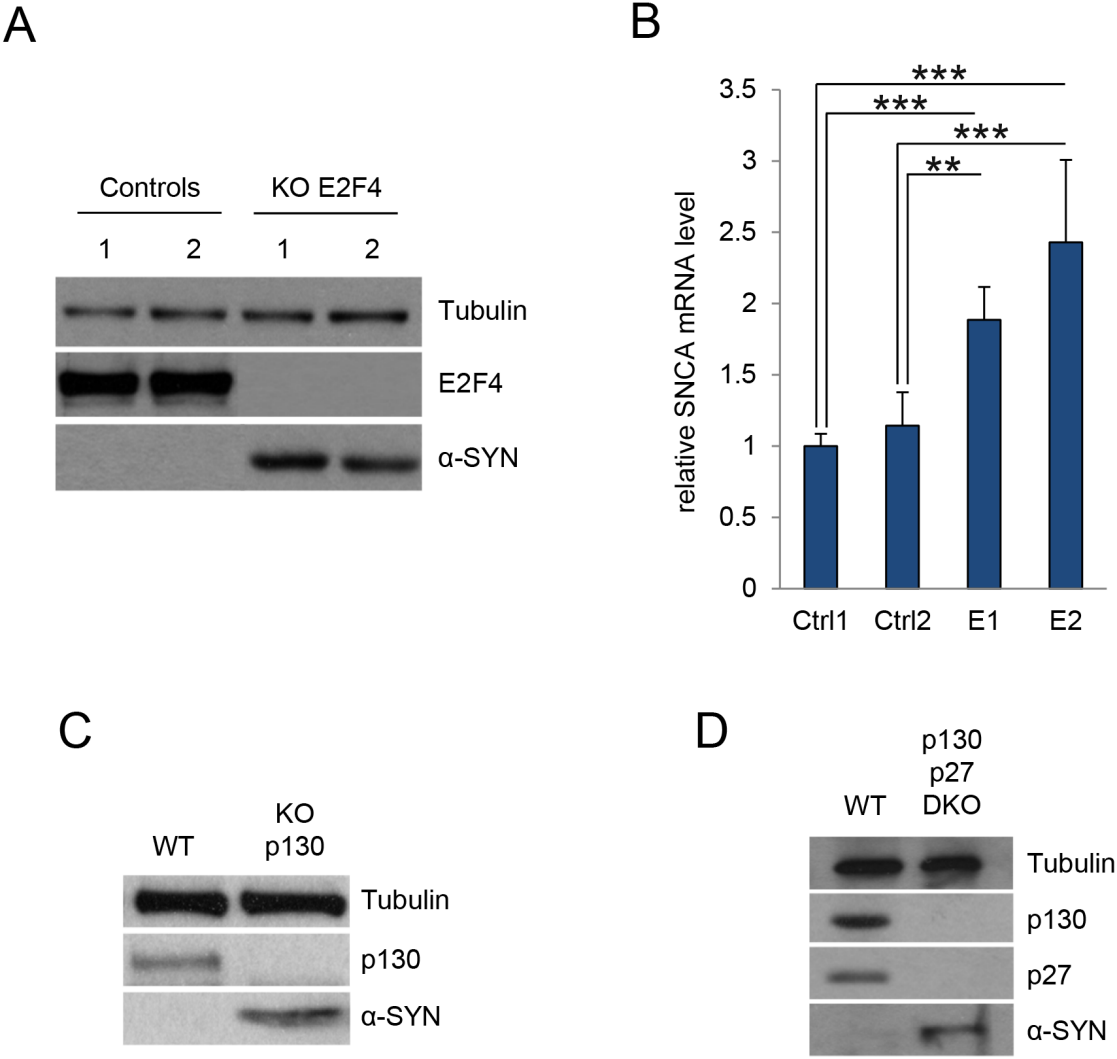
E2F4 and p130 regulate α-SYN expression **(A)** Control MEFS and E2F4 KO MEFs were assessed for E2F4 and α-SYN by WB. Tubulin was used as a loading control. **(B)** α-SYN mRNA levels were determined by qPCR in the same cells as in (A). Results are expressed relative to Control 1 (Ctrl1). **(C)** Control MEFS (WT) and p130 KO MEFs were assessed for p130 and α-SYN by WB. Tubulin was used as a loading control. **(D)** Control MEFS (WT) and double KO MEFs p130/p27 (DKO) were assessed for p130, p27 and α-SYN by WB. Tubulin was used as a loading control. In all experiments results are the mean value ± SD of at least three independent experiments. Statistical analyses were performed using the Student’s t-test. ^**^P<0.01 and ^***^P<0.001.

As p27 and p21 collaborate in the transcriptional regulation of p130/E2F4 repressed genes [15], we aimed to analyze the possibility that p27 and p21 regulate α-SYN expression trough the participation of p130/E2F4 complexes. Thus, we first determined the levels of α-SYN in E2F4KO MEFs finding elevated α-SYN protein (Figure 3A) and mRNA (Figure 3B) in the absence of E2F4. The increase in α-SYN protein was also observed when E2F4 was knocked down in a different cellular type, as 293T (Supplementary Figure 1). Since E2F4 loading on its target promoters requires the presence of p130, we also analyzed the α-SYN levels in p130KO MEFs. As shown in Figure 3C, we observed an increase in α-SYN expression in these cells. Additionally, we found that α-SYN increased in double KO (p130 and p27) MEFs (Figure 3D).

### p27, p21 and E2F4 associate with specific regions of the *Snca* gene promoter and regulate its transcription

**Figure 4:**
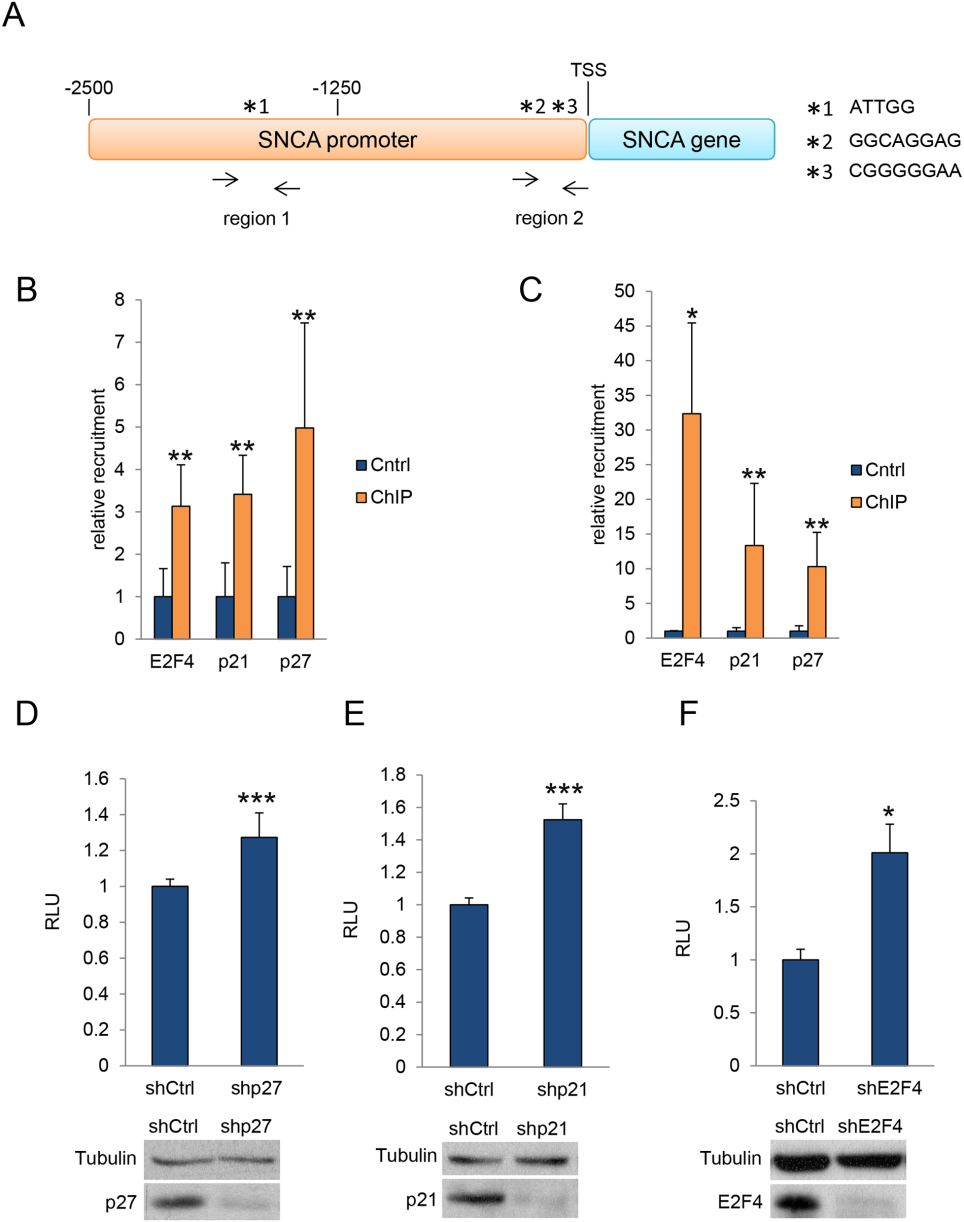
ChIP and luciferase assays **(A)** Representation of the putative E2F4 binding sites in the SNCA promoter. Two regions (region 1 (from -2200 to -1350 bp) and region 2 (from -300 bp to TSS)) on the proximal α-SYN promoter contain putative E2F4 consensus sequences. Sequence ^*^1 was located in region 1 whereas sequences ^*^2 and ^*^3 were located in region 2. **(B)** E2F4, p21 and p27 association with Region 1 of the SNCA promoter as determined by ChIP in c17.2 cells. **(C)** E2F4, p21 and p27 association with Region 2 of the SNCA promoter as determined by ChIP in c17.2 cells. **(D)** Expression of luciferase reporter gene in 293T cells infected with ShRNA control or ShRNA for p27. A WB showing the levels of p27 is shown in the bottom panel. **(E)** Expression of luciferase reporter gene in 293T cells infected with ShRNA control or ShRNA for p21. A WB showing the levels of p21 is shown in the bottom panel. **(F)** Expression of luciferase reporter gene in 293T cells infected with ShRNA control or ShRNA for E2F4. A WB showing the levels of E2F4 is shown in the bottom panel. In all experiments results are the mean value ± SD of at least three independent experiments. Statistical analyses were performed using the Student’s t-test. ^*^P<0.05, ^**^P<0.01 and ^***^P<0.001.

To analyze whether p27 directly regulates α-SYN expression, we first identified, by *in silico* analysis (bioinformatics tools from FIMO web site), specific E2F4 consensus sequences on the α-SYN promoter. As observed in Figure 4A, two regions (region 1 (from -2200 to – 1350 bp) and region 2 (from -300 bp to TSS)) on the proximal α-SYN promoter contain putative E2F4 consensus sequences. Sequence ^*^1 was located in region 1 whereas sequences ^*^2 and ^*^3 were located in region 2. We designed specific primers to detect these regions after ChIP analysis (Supplementary Table 1). ChIP was performed in c17.2 cells using specific anti-E2F4, anti-p27 and anti-p21 antibodies. ChIP with no antibodies was used as a control. The specific association of E2F4, p27 and p21 to regions 1 and 2 was detected by qPCR using the designed primers. We observed that E2F4 significantly associates with region 1 (Figure 4B) and region 2 (Figure 4C). Interestingly, both p27 and p21 can be also found to associate with the same regions of the α-SYN promoter (Figure 4B and 4C). These results clearly indicate that p27 and p21 can associate to these regions through its interaction with E2F4, as previously shown in other gene promoters [11, 15].

Finally, we also performed luciferase assays as a functional demonstration of the interaction of p27, p21 and E2F4 with the α-SYN promoter. To do so, we transfected 293T cells with a vector containing a luciferase reporter gene under the control of the α-SYN promoter. Additionally, we knocked down p27, p21 or E2F4 using specific shRNAs and subsequently quantified luciferase expression. Results indicated that elimination of p27 (Figure 4D), p21 (Figure 4E) or E2F4 (Figure 4F) significantly induced the expression of the α-SYN –driven luciferase reporter. These results confirm that α-SYN transcription is actively repressed by p27/p21/E2F4 complexes, revealing a specific role of p27 (with the collaboration of p21) in the regulation of α-SYN expression.

### Increased levels of α-SYN in cerebellar cells of p27 KO mice

**Figure 5:**
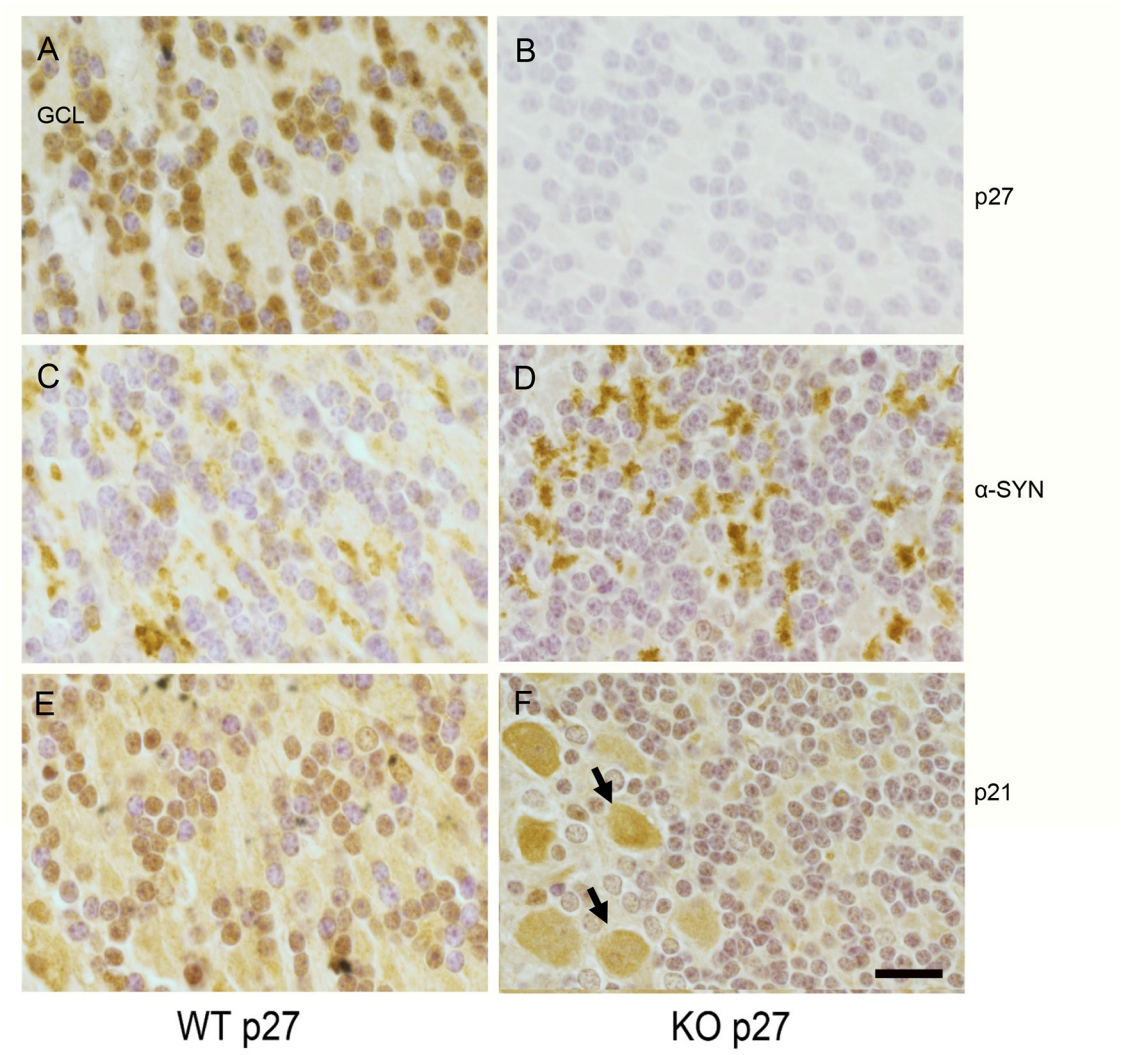
p27, p21 and α-SYN levels in cerebellar sections from WT and p27KO mice Distribution of p27, p21 and α-SYN was visualized by immunohistochemistry in cerebellum sections from WT and p27KO mice. **(A)** p27 stained sections from WT animals. Nuclear staining of p27 can be observed in the granule cell layer (GCL). **(B)** p27 stained sections from p27KO mice. **(C)** α-SYN staining in sections from WT mice. **(D)** α-SYN staining in sections from p27KO mice. **(E)** p21 stained sections from WT animals. **(F)** p21 stained sections from p27KO animals. Arrows indicate Purkinje cells. Scale bar = 10 μm.

In light of our molecular data, we next decided to evaluate whether we could observe any inverse correlation between p27 and α-SYN levels in the adult brain. We selected the cerebellum because it has been described that Purkinje and granule cells have detectable levels of p27 [23]. Accordingly, immunohistochemistry with specific p27 antibodies performed on WT murine adult cerebellar tissue showed that most of the neurons in the granule cell layer contain high amounts of nuclear p27 (Figure 5A and Supplementary Figure 2A). We confirmed the specificity of the staining using p27-KO tissue as a control (Figure 5B and Supplementary Figure 2A). Since p27 and p21 collaborate in the transcriptional regulation of several genes [15] and it has been shown in MEFs that p21 expression might be repressed by p27 [18], we also performed immunohistochemistry using anti-p21 antibodies in cerebellar sections from WT and p27KO mice. In the absence of p27, p21 levels were increased in Purkinje neurons but interestingly not in granule cells (Figure 5E, 5F and Supplementary Figure 3). These results indicate that, rather than being a general phenomenon, the induction of p21 in cells lacking p27, seems to be cell type-specific.

In this scenario, we analyzed the levels of α-SYN in the granule cell layer in both WT and p27-null tissue. Our results reveal that, contrary to the pattern found on WT tissue (Figure 5C), increased levels of α-SYN aggregates can be clearly detected in cells from the p27KO sections (Figure 5D). It can also be observed that these aggregates are located in non-nuclear domains (Figure 5D). The specificity of the α-SYN antibodies was confirmed using cerebellar sections of α-SYN KO animals (Supplementary Figure 2B). These results support a potential role of p27 and p21 in the regulation of α-SYN expression.

## DISCUSSION

Our results identify p27 as a transcriptional regulator of α-SYN expression. The data add to previous results demonstrating a role for p27 in gene expression aside its canonical role as Cdk-inhibitor. Furthermore, our finding that α-SYN expression is under the control of p27, also *in vivo*, suggest an intriguing novel connection between p27 and PD that may reveal p27 as a potential therapeutic target.

Synucleinopathies are neurodegenerative diseases characterized by the abnormal accumulation of aggregates of α-SYN protein in neurons, nerve fibers or glial cells [24].

There are three main types of synucleinopathy: Parkinson's disease (PD), dementia with Lewy bodies, and multiple system atrophy. Other rare disorders, such as various neuroaxonal dystrophies, also have α-synuclein pathologies.

PD is a common neurodegenerative disorder that affects 2–3% of the population ≥65 years of age [25]. The characteristic features of PD include neuronal loss in the *substantia nigra pars compacta* which causes striatal dopamine deficiency and intracellular inclusions containing aggregates of α-SYN. In PD patients, these α-SYN inclusions (Lewy bodies) are found in certain neurons of different regions of the brain [26, 27]. In heritable forms of this disease, mutations in the α-SYN gene (SNCA) have been observed and this observation supports the important role of this protein in PD [28, 29].

The functional roles of α-SYN are not well characterized but it is mainly located in the presynaptic terminals of neurons, although it can be nuclear or it may be also in the mitochondria. It has been involved in synaptic vesicle dynamics, mitochondrial function and in intracellular trafficking [29]. α-SYN acquires neurotoxic properties since the soluble monomers can form oligomers and subsequently large fibrils that finally aggregate to make up the Lewy bodies [30, 31]. The determinants of this aggregation can be multiple: overproduction of the protein, the presence of mutations or impairment of the mechanisms involved in its degradation [25]. As mutations of SNCA are observed only in a reduced number of PD patients [32], in most of the non-heritable forms of PD (sporadic PD) the accumulation of α-SYN by overproduction or by impairment of the degradative mechanisms are the most significant causes of the generation of Lewy bodies.

Despite the relevance of α-SYN in PD very few is known about the mechanisms involved in the regulation of its expression. There only few reports focused in the regulation of α-SYN transcription. Specifically, it has been shown that four TFs, namely C/EBPβ, GATA-1/2, ZSCAN 21 and ZNF 219 can associate with regions of the intron 1 of SNCA gene thus activating its expression [33–36]. More recently, it has been shown that the association of p53 to the SNCA promoter induces the expression of α-SYN [37]. Despite of this promising advances, the putative role of α-SYN transcriptional regulation by these TFs in PD induction and propagation still remains to be clarified. Our findings describe for the first time a mechanism of repression of SNCA. Specifically, we report here that neural c17.2 - p27KO cells expressed higher levels of α-SYN mRNA than controls, suggesting that p27 might be involved in negative transcriptional regulation of α-SYN. We confirmed that the observed increase of α-SYN, in these p27KO cells, depends on p27 by showing that the re-introduction of p27 in these cells induces a decrease of α-SYN. We further supported this evidence by demonstrating the association of p27 with two specific regions of the SCNA gene promoter and by luciferase assays in which luciferase expression is under the control the α-SYN promoter. These luciferase experiments demonstrated that the decrease of p27 induced α-SYN expression. Finally, further support was provided by the observation that α-SYN accumulates in the granular cell layer of the cerebellum from p27KO mice. The origin of this α-SYN is still unknown.

We next explored whether p21 collaborates with p27 in the transcriptional regulation of α-SYN expression. We previously reported that p27 indirectly regulates p21 expression in MEFs. Specifically, we demonstrated that p27 represses the expression of Pitx2, a transcriptional activator of p21. However, our observation that in cerebellar sections of p27KO mice p21 only increases in Purkinje cells but, on the contrary, it decreases in the granule cell layer, indicates that this p21 regulation by p27 is cell-type depending but not a general phenomenon. We describe here that knocking down p21 induces the increase of α-SYN expression not only in control cells but also in p27KO clones. These collaboration between p27 and p21 in the regulation of α-SYN expression strongly supported that this gene could be repressed by p130/E2F4 complexes. We confirmed this possibility by demonstrating that in both E2F4- or p130-KO cells a clear increase of α-SYN levels was induced.

Interestingly, ChIP analysis revealed that p21 and E2F4 also associate with the same two specific regions of the α-Syn promoter that also interact with p27. Finally, luciferase assay confirms that p21 and E2F4 also directly regulate α-SYN transcription.

Thus, our results describe the mechanism by which p27 in collaboration with p21 regulates the expression of α-SYN through the interaction with p130/E2F4 repressor complexes as it has been reported for cell cycle genes but also for other no cell cycle related genes as Sox2. Interestingly, this regulatory mechanism also involves different cyclin-Cdk complexes that would phosphorylate p130 [15]. We report here that Cdk inhibition by Roscovitine induced a decrease of α-SYN expression indicating a role of Cdks in this regulation. All these results reveal that p27 in collaboration with p21 regulates α-SYN expression by a mechanism involving p130/E2F4 repressive complexes and cyclin-Cdks, according to a model previously reported by our group [15].

The description of this mechanism is especially relevant in the context of synucleinopathies and opens the possibility of a participation of p27 in the induction and propagation of these diseases. Specifically, it should be of interest to analyze whether in the sporadic forms of PD or in the cerebellar subtype of the multiple system atrophy, formation of α-SYN aggregates could be a consequence of a progressive decrease of p27 levels in the affected cells. This possibility is compatible with results reported here indicating that the decrease of p27 induce an elevated expression of α-SYN.

In summary, we describe here for the first time a mechanism involved in the negative regulation of α-SYN expression. This regulation is performed by p27 in collaboration with p21 and mediated by p130/E2F4 repressor complexes. These results allows to postulate that this regulatory mechanism can be disrupted or altered in the process of induction or/and propagation of PD and other synucleinopathies. Thus, α-SYN accumulation, aggregation and toxicity in patients affected of any synucleinopathy could putatively be associated with a progressive alteration of this regulatory mechanism.

## MATERIALS AND METHODS

### Cell culture and transfection

HEK-293T cells (from ATCC), c17.2 cells (a kind gift of Dr. Evan Snyder), p130KO and double p130/p27 KO MEFs- (a kind gift from Dr Anxo Vidal), p27KO, p21 KO and E2F4 KO MEFs were cultured in Dulbecco’s modified Eagle Medium supplemented with 10% fetal bovine serum, 5% Glutamine and 5% Penicillin/Streptomycin. Cultures were maintained at 37°C and 5% CO_2_. Plasmids were transfected in HEK-293T cells using lipofectamine 2000 (Invitrogen) following manufacturer’s instructions. The Cdk inhibitor Roscovitine (Sigma) was added to the culture at 20 μM final concentration. All cells were free from mycoplasma.

### Animals

All animal experiments were performed in accordance with the Guidelines of the European Union Council (86/609/EU) and Spanish Government (BOE 67/8509-12) and approved by the Ethics and Scientific Committees of the Spanish National Research Council (CSIC) and the University of Barcelona. All the protocols used were registered at the *“Departament d’Agricultura, Ramaderia, Pesca, Alimentació i Medi Natural de la Generalitat de Catalunya”*. Mice were maintained under regulated light and temperature conditions at the animal facilities of the Faculty of Medicine, University of Barcelona. All efforts were made to minimize animal suffering and discomfort and to reduce the number of animals used. Under deep anesthesia with pentobarbital, adult mice were intracardially perfused with phosphate-buffered saline (PBS) followed by 4% paraformaldehyde in 0.1 M phosphate buffer (PB).The brains were removed and post-fixed in the same solution overnight at 4°C. After several washes with PB, samples were processed and dehydrated through a series of graded ethanol to xylene and infiltrated with paraffin. Serially sectioned sagittal sections (5 μm) were obtained using a Microtome. The sections were mounted on gelatin-coated slides.

### Immunohistochemistry

After dewaxing with xylene and rehydrated with graded ethanols, sections were pretreated in an autoclave for 15 min at 120°C in antigen retrieval reagent-basic (R&D, CTS013). Following incubation, samples were cooled to room temperature (approximately 5-10 min). After one wash in PBS, sections were permeabilized with three washes (5 min each) of 0.3% Triton X-100 in PBS (PBST). Then, they were treated with H_2_O_2_ (3%)-methanol to inhibit endogenous peroxidases and subsequently incubated with 10% normal serum in PBST during 2 h to prevent non-specific binding. Finally, sections were incubated overnight at 4°C with primary antibodies: mouse monoclonal anti-p27 (1/50; BD Biosciences, 610242); mouse monoclonal anti-p21 (1/50, Santa Cruz, Sc-6246) and rabbit polyclonal anti-α-SYN (1/250, Santa Cruz, Sc-7011). After washing, sections were reacted with biotinylated-conjugated secondary antibodies (1/200 Vector, biotinylated anti-mouse, Cat. BA-2000 or biotinylated anti-rabbit, BA-1000) using an ABC peroxidase staining kit (Thermo Scientific, 32020). Diaminobenzidine was used as the chromogen. Sections were counterstained with hematoxylin. After one wash with PBS, they were dehydrated in graded alcohol to xylene and mounted with DePeX. Negative controls: the specificity of p27 and α-SYN primary antibodies was verified using the appropriate KO mice. The specificity of p21 primary antibody was verified in a previous work [38]. Microscopy images were obtained with an Eclipse 1000 Nikon microscope (Nikon, Tokyo, Japan) and a digital camera (Olympus DP72, Tokyo, Japan).

### Gel electrophoresis and Western blotting

Samples were loaded on sodium dodecyl sulphate polyacrylamide gel electrophoresis (SDS-PAGE) gels. After electrophoresis, gels were transferred to Hybond-C (Amersham Life Sciences) membranes. Then, transferred proteins were subjected to western blotting (WB) using the following antibodies: anti-p21 (sc-6246) and anti-E2F4 (sc-866) from Santa Cruz, anti-p27 (610242) from BD Transduction Laboratories, anti- α-SYN (610787) from BD Biosciences and anti-tubulin (T9026) from Sigma Aldrich.

### Chromatin immunoprecipitation

c17.2 cells were grown in an 80% confluence plate. Chromatin immunoprecipitation (ChIP) assays were performed as previously described [39]. Briefly, cells were lysed and chromatin from crosslinked cells was sonicated. Chromatin was incubated with 5 μg of anti-p27 (C-19, Santa Cruz), anti-p21 (sc-397, Santa Cruz) or anti-E2F4 (sc-866, Santa Cruz) in RIPA buffer (50 mM Tris - HCl pH 7.5, 150 mM NaCl, 1% NP-40, 0.5% Sodium deoxycholate, 0.1% SDS, 1 mM EDTA, 1 mM DTT, 1 mM PMSF, 0.1 mM Na_3_VO_4_, 0.5 μg/μl aprotinin, 10 μg/μl leupeptin) adding 20 μl of Magna ChIP Protein A magnetic beads (Millipore). Samples were incubated in rotation overnight at 4°C. Samples incubated without antibodies were used as a control. Beads were washed with low-salt buffer, high-salt buffer, LiCl buffer and TE buffer. Subsequent elution and purification of the immunoprecipitated DNA-protein complexes was performed using the IPure kit (Diagenode) according to manufacturer’s protocol. Samples were analysed by qPCR. Primer sequences used for qPCR of SNCA promoter genomic regions are listed in Supplementary Table 1.

### RNA extraction, reverse transcription-PCR and qPCR for gene expression analysis

Total RNA from cells was extracted using High Pure RNA Isolation kit (Roche). cDNA was obtained from 1 μg of RNA using SuperScript ViLO cDNA synthesis (Invitrogen) according to manufacturer’s instructions. Gene expression was analysed by real-time PCR using LightCycler 480 SYBR green I master mix (Roche), corrected by GAPDH expression and expressed as relative units. Primer sequences used for qPCR assessment of SNCA, p27, p21 and control genes are listed in Supplementary Table 1.

### Protein overexpression and shRNA lentiviral infection

HEK-293T and c17.2 cells were infected with pLV-Ires-GFP plasmid expressing the p27 WT gene. HEK-293T cells were infected with MISSION shRNA control vector and specific p27, p21 or E2F4 shRNAs (Sigma Aldrich) with the following sequences:

p27: 5′-CCGGGCGCAAGTGGAATTTCGATTTCTCGAGAAATCGAAATTCCACTTGCGCTTTTTG- 3′.
p21: 5′-CCGGCTATCACTCCAAGCGCAGATTCTCGAGAATCTGCGCTTGGAGTGATAGTTTTTG-3′.
E2F4: 5’-CCGGGCAAGAACTAGACCAGCACAACTCGAGTTGTGCTGGTCTAGTTCTTGCTTTTTG-3’

The protocol for viral particles production and cell infections has been described elsewhere [40]. 24 h after infection, cells expressing shRNAs were selected with 2 mg/ml of Puromycin (Sigma Aldrich) for 5 days. ShRNA-mediated down-regulation was tested by WB with specific antibodies.

### CRISPR transfection and clone production

4 μg of control double Nickase plasmid (sc-437281) or 4 μg of double Nickase plasmid expressing a guide RNA sequence targeting a region on p27 gene (sc-419608-NIC) were used to transfect c17.2 cells. 48 h after transfection, GFP-positive cells were sorted using a BD FACS Aria Cell Sorting System, selecting one single cell per well. 2-3 weeks later, clones were amplified and p27 expression was analysed by WB.

### β-galactosidase and luciferase assays

Luciferase vector was obtained by cloning a specific region from the SNCA promoter (-2000 pb) into a pGL2 Basic vector. Primers for the selected gene were designed by adding MluI and BglII target sequences at 5′ and 3′, respectively. The primers used for this amplification were: SNCA promoter Forward 5′ - CTAGAAGGAGAGAAGTCGATAGTG- 3′, SNCA promoter Reverse 5′ - GGAGCACATTCCCCCGGA TGGAAG- 3′.

Amplification of SNCA promoter sequence was made by PCR using genomic DNA and cloning the PCR products into a pGL2 vector. HEK-293T cells expressing shRNA control, shRNA for p27 or shRNA for E2F4 were co-transfected with CMV-βGal vector and a vector containing SNCA promoter. β-galactosidase and luciferase assays were performed 48 h after transfection. β-galactosidase activity was detected using ONPG (Sigma) and read at 405 nm wavelength. Luciferase assays (Luciferase Assay System; Promega, Madison, WI) were performed following manufacturer’s instructions. Luciferase/β-galactosidase ratio was performed and shown as arbitrary units (RLU: relative light unit).

### Statistical analysis

GraphPad Prism 5.01 (GraphPad Software, San Diego, CA, USA) was used for data analysis. The Student’s t-test was applied to determine significant differences between groups. In these analyses P-values of 0.05 were considered to be significant. At least three independent samples were analysed in each experiment. The significance of the correlation between p27 and p21 levels was estimated by linear regression analysis using the statistical package for the social sciences (SPSS).

## SUPPLEMENTARY MATERIALS FIGURES AND TABLE


