# Supplementary material for: p27Kip1 regulates alpha-synuclein expression
Source: Oncotarget. 2018 Mar 27;9(23):16368–79. doi: 10.18632/oncotarget.24687 (PMC5893246; doi:10.18632/oncotarget.24687)
Supplement: Supplementary file 1 [file oncotarget-09-16368-s001.pdf]

p27^{Kip1} regulates alpha-synuclein expression

SUPPLEMENTARY MATERIALS

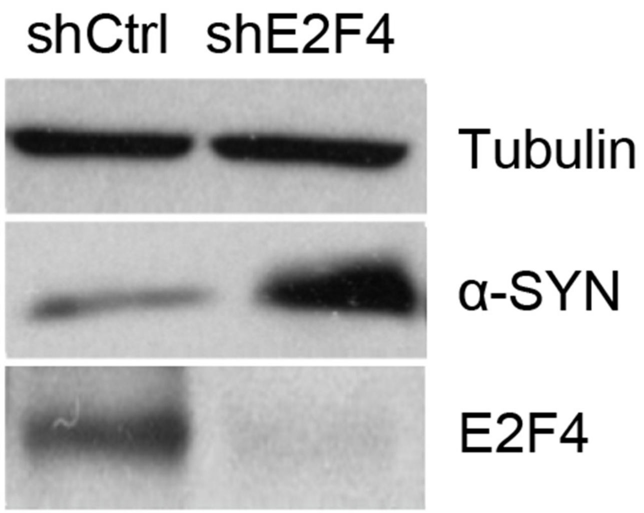

Supplementary Figure 1: HEK-293T cells infected with ShRNA control or ShRNA for E2F4 were assessed for E2F4 and α-SYN protein by WB. Tubulin was used as a loading control.

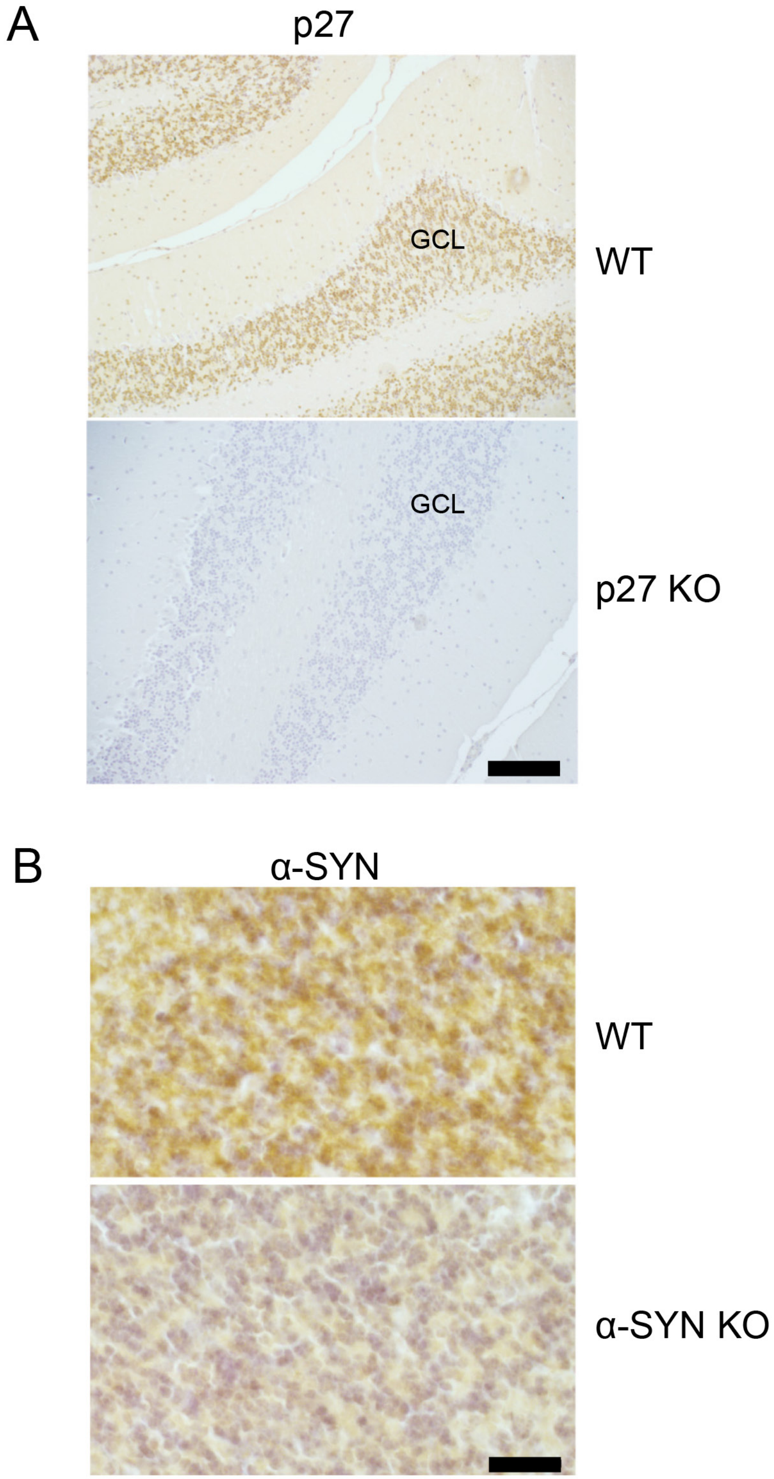

Supplementary Figure 2: (A) Specificity of p27 antibody was determined by immunohistochemistry in cerebellum sections from WT and p27 KO mice. Scale bar = 20 µm. **(B)** Specificity of α-SYN antibody was determined by immunohistochemistry in cerebellum sections from WT and α-SYN KO mice. Scale bar = 10 µm. GCL: Granule cell layer.

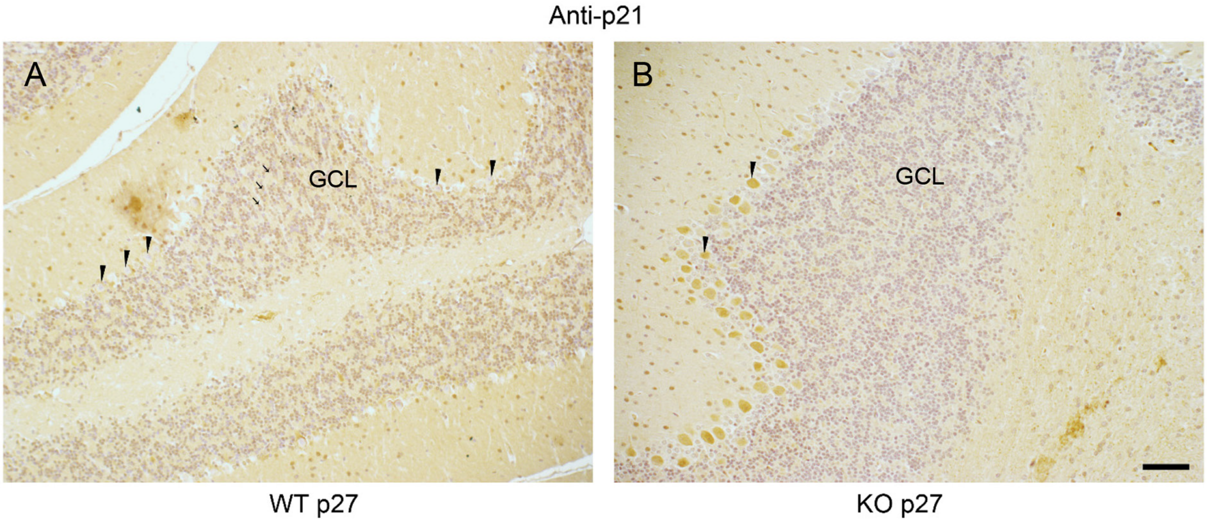

Supplementary Figure 3: Distribution of p21 was visualized by immunohistochemistry in cerebellum sections from WT and p27KO mice. (A) p21 stained sections from WT. **(B)** p21 stained sections from p27 KO mice. Scale bar = 20 µm. Arrows indicate cells from the granule cell layer (GCL). Arrow heads indicate Purkinje cells.

Supplementary Table 1: List of primers used for qRT- PCR and for ChIP

q-RT-PCR primers	sequences
p27 forward	ATCTGCCTCTAAAAGCATTG
p27 reverse	CCATCCCTAACGTTTATGTG
p21 forward	GTACTTCCTCTGCCCTGCTG
p21 reverse	TCTGCGCTTGGAGTGATAGA
SNCA forward	GGTTCCAAAACTAAGGAAGG
SNCA reverse	CCTCCAACATTTGTCACTTG
GAPDH forward	ACCCAGAAGACTGTGGATGG
GAPDH reverse	ACACATTGGGGGTAGGAACA

ChIP primers	sequences
region 1 forward	CAGGCCTGGCAAAACATTAT
region 1 reverse	AGGCTTCAGCCTTTTCCTTC
region 2 forward	CTCTCTGGTGTGGCATCTGG
region 2 reverse	GTGAGGGCTGTGGGTATCTG
